# Characterization of Epoxy-Based Rapid Mold with Profiled Conformal Cooling Channel

**DOI:** 10.3390/polym14153017

**Published:** 2022-07-26

**Authors:** Chil-Chyuan Kuo, Yi-Jun Zhu

**Affiliations:** 1 Department of Mechanical Engineering, Ming Chi University of Technology, No. 84, Gungjuan Road, New Taipei City 243, Taiwan; m09118003@mail.mcut.edu.tw; 2 Research Center for Intelligent Medical Devices, Ming Chi University of Technology, No. 84, Gungjuan Road, New Taipei City 243, Taiwan

**Keywords:** cooling channel, wax injection mold, epoxy-based injection mold, cooling time, material formulation, profiled CCC

## Abstract

Based on the experience of the foundry industry, reducing the demolding time is the key for mass production of wax patterns with sophisticated geometries. Integration of numerical simulation and rapid tooling technology for decreasing the time to market is essential in advanced manufacturing technology. However, characterization of epoxy-based rapid molds with a profiled conformal cooling channel (PCCC) using computer-aided engineering simulation of the epoxy-based rapid mold with PCCC was not found in the literature. In this study, epoxy-based rapid molds with PCCC were characterized numerically and experimentally. The cooling performance of wax injection molds with two different kinds of cross-sections of the cooling channel was investigated. Four pairs of injection molds with PCCC were implemented using four different kinds of material formulations. It was found that the cooling performance of the PCCC was better than a circular conformal cooling channel (CCCC) since the PCCC maintained a more uniform and steady cooling performance of injection-molded product than CCCC. Epoxy resin added with 41 vol.% Cu powder seems to be a cost-effective empirical material formulation in terms of cooling time and material costs. This empirical material formulation provided an injection mold with low material cost and good cooling performance simultaneously compared to an injection mold fabricated with commercial material. The cooling performance could reach 88% of that of the injection mold fabricated with commercial material. The material cost of making the injection mold was only about 60% of that of the injection mold fabricated with commercial material. The coolant flow rate had no significant effect on the cooling time, whereas the cooling time of the wax pattern was affected by coolant temperature significantly.

## 1. Introduction

Investment casting (IC), also called lost-wax casting, has been widely used to manufacture metal components with complex geometries. Dong et al. [1] looked into the simulation of shrinkage during IC for regular structures of hollow turbine blades. Results revealed that the simulated and measured shrinkages have the same trend. Average deviations between the predicted and measured values of the transitional and normal sections were about 5.8% and 2.4%, respectively. Wang et al. [2] proposed a data-driven framework to study the optimum gating system using an optimization algorithm of machine learning. Results showed that that optimization of a gating design is promising with high accuracy and high efficiency. The casting yield was increased by approximately 14.91%. The technical term conformal denotes that the geometry of the cooling channels follows the mold surface geometry. In the IC industry, the production rate of wax patterns was not acceptable for a wax injection molding tool with a straight-drilled cooling channel. To overcome this drawback, the conformal cooling channel (CCC) was proposed to improve both productivity and quality of the molded parts [3]. Muvunzi et al. [4] designed a hot stamping tool with CCC. Design guidelines were also proposed for an additive manufacturing-based conformal cooling system for hot stamping tools. Results revealed that a molded part with the desired hardness could be manufactured under a cooling time of 5 s. Brigden [5] designed the conformal cooling layers using the method of self-supporting lattices. Results showed that the cooling time of the plastic product could be reduced by approximately 26.34%. Vojnova [6] analyzed the advantages of an injection mold with CCC after plastic injection molding. Results suggested that CCC is suitable for geometrically complicated molds to remove the heat generated in the hot spots. Kitayama et al. [7] looked into process parameters of plastic injection molding for reducing the warpage of plastic products using CCC. This work confirmed that the CCC provides a reduction in the warpage of plastic products. Wang et al. [8] proposed a plastic injection mold with a spherical spiral conformal cooling system. Results suggested that defects of plastic products, such as residual stress or warpage, cannot be ignored. Chen et al. [9] developed segmented finite element models for optimizing geometries of the conformal cooling system. Results suggested that the saving in computational time was about 92.6% compared to the entire model of the U-shape component. Singraur et al. [10] focused on the advancements in design and fabrication of CCC for improvement in the injection molding process. This paper also reviewed the manufacturing processes for fabricating CCCs employed in the injection molds. Abbes et al. [11] looked into an injection molding tool with CCC to increase productivity. This work found that the injection cycle time could be reduced up to 65.6% using a hybrid mold with CCC. Li et al. [12] used a topology method to optimize CCC. This work suggested that the proposed method could realize the optimization of the CCC. Mercado-Colmenero et al. [13] employed an innovative lattice element of CCC for an injection mold. It was found that the new cooling lattices improved the efficiency of thermal exchange in the cooling stage of the molded products with large concavities, fine details, internal turrets, and housings. Marin et al. [14] designed a new design conformal cooling channel for injection molding. Results found that the proposed hybrid manufacture of the inserts reduced the manufacturing costs and time by 53% and 60%, respectively. Generally, the design and optimization of the CCC is a time-consuming process. Computer-aided engineering simulation is effective for designing the conformal cooling channels. Altaf et al. [15] proposed a technique of fabricating profiled conformal cooling channels (PCCCs) in an aluminum-added epoxy mold using both rapid prototyping and rapid tooling techniques. Experimental results revealed that an injection mold with PCCC had less cooling time than an injection mold with a circular conformal cooling channel (CCCC).

In modern foundry, the productivity of wax patterns was not good enough for a wax injection molding tool with conventional cooling channels machined in straight lines. The design of the cooling system has a major effect on the cooling performance of the injection mold. Naturally, the shape and layout of the cooling channel have a great influence on the cooling performance of the product. Therefore, designing a cooling system for a new wax injection mold has been a very important step in the investment casting industry since the cooling time is regarded as a key factor influencing the throughput during the mass production of wax patterns using the wax injection molding process. The epoxy-based rapid mold provides promising advantages in shortening the cost and lead time for mold making. Based on the practical experience, the cooling system in the mold plays an important role in the wax injection molding process because the throughput of wax patterns is influenced by the cooling performance greatly. However, a critical issue overlooked in the literature on CCC is the details regarding the computer-aided engineering simulation of the epoxy-based rapid mold with PCCC. Conventionally, the CCC is typically designed by a trial-and-error method based on experience. In this study, the difference in the effectiveness between PCCC and CCCC was verified via numerical simulation. In addition, the cost issue restricts the use of a conventional steel mold in the research and development stage of a new product. To overcome this problem, the epoxy-based rapid mold was introduced. Four wax injection molds were fabricated using four different kinds of epoxy-based rapid tooling materials. In general, the design and optimization of conformal cooling channels is a time-consuming step. To overcome this drawback, the aim of this work was to characterize the epoxy-based rapid mold with PCCC and investigate the difference between CCCC and PCCC using the numerical analysis in advance. The cooling performance of the wax injection mold was then evaluated using the wax injection molding process and compared to simulation results. Finally, a cost-effective empirical material formulation for making wax injection molds with high cooling performance was proposed.

## 2. Experimental Details

**Figure 1:**
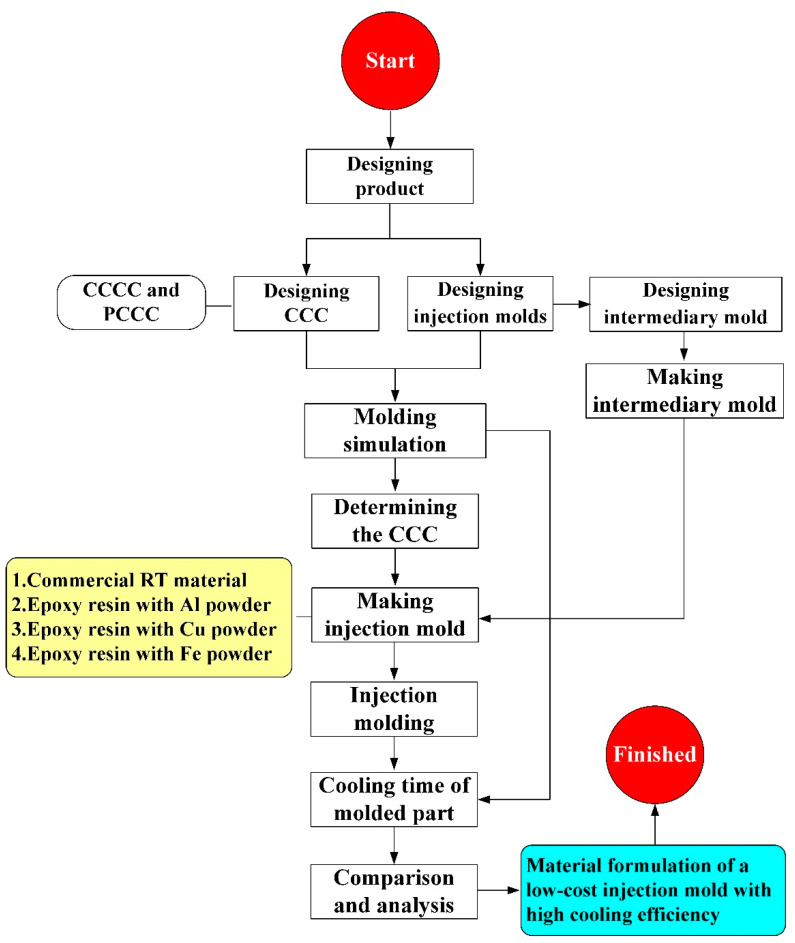
The detailed steps of the research process of this study.

**Figure 2:**
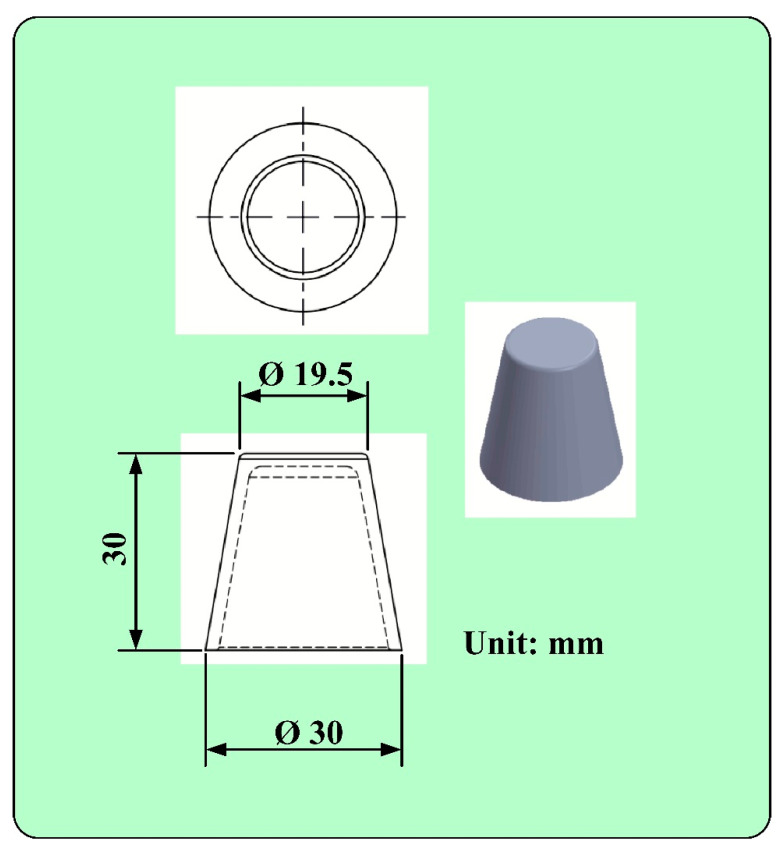
3D CAD model of the wax pattern.

**Figure 3:**
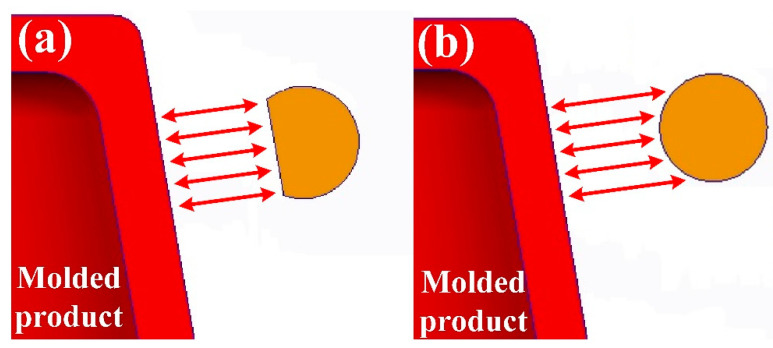
Schematic illustrations of the cross-section of (**a**) PCCC and (**b**) CCCC.

**Figure 4:**
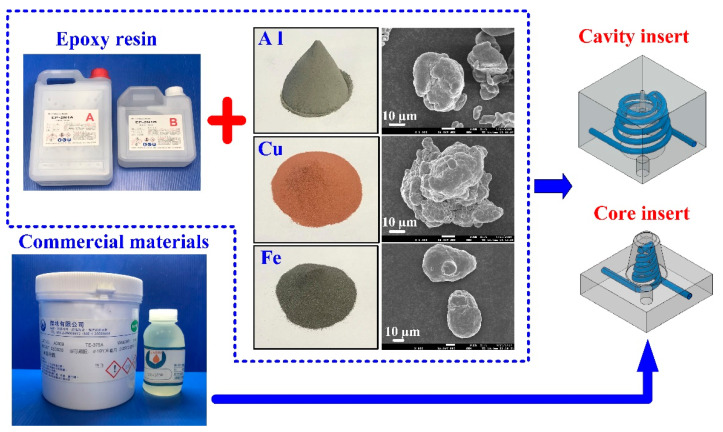
Three different kinds of fillers were added into the epoxy resin to make injection molds.

Figure 1 shows the detailed steps of the research process of this study. The geometric models of the rapid mold, wax pattern, and cooling system were designed using computer-aided design software (Cero, parametric technology corporation Inc., Taipei, Taiwan). Figure 2 shows the three-dimensional (3D) computer-aided design (CAD) model of the wax pattern. The maximum outer diameter, minimum outer diameter, height, and thickness were 30 mm, 19.5 mm, 30 mm, and 2 mm, respectively. There are eight cross-sections of CCC [16], i.e., circular, rectangular, elliptical, square, semicircular, profiled, fluted, and water drop. In this study, two kinds of cross-sections of the CCC were used to study. Figure 3 shows the schematic illustrations of both CCCC and PCCC. Particularly, the distance between the edge of the PCCC and the wall of the mold cavity is microscopically equidistant. Conversely, the distance between the edge of the CCCC and the wall of the mold cavity is microscopically non-equidistant. Figure 4 shows the three different kinds of fillers that were used in this study. The curing agent (EP-2N1, Ruixin Inc., Taipei, Taiwan) and base compound of the epoxy resin (EP-2N1, Ruixin Inc., Taipei, Taiwan) were mixed to prepare matrix materials in advance. In this study, three different kinds of fillers (First chemical Inc., Taipei, Taiwan), i.e., aluminum (Al) powder, copper (Cu) powder, and iron (Fe) were added into the matrix materials to make injection molds. Three different kinds of powders were examined by field-emission scanning electron microscopy (FE-SEM) (JEC3000-FC, JEOL Inc., Tokyo, Japan). The average particle sizes of Al, Fe, and Cu powders were 48 µm, 48 µm, and 45 µm, respectively, with a purity of about 96–99%. The average viscosity of the epoxy resin was about 13,000 mPa-s. In this study, a water cup was selected as a molded part. The top edge of the product was set as parting line. Therefore, the core and cavity inserts could be determined easily. In addition, the geometries of the CCC for core and cavity inserts were different. To evaluate the economic benefit of the optimum material recipe developed in this study, the commercial material (TE-375, Jasdi Chemicals, Inc., New Taipei City, Taiwan) was also employed to fabricate injection molds. To study the uniformity of the added powder inside the rapid mold, the chemical compositions of the top, middle, and bottom were examined using an energy-dispersive X-ray spectroscopy (EDS) (D8 ADVANCE, Bruker Inc., Billerica, MA, USA).

**Figure 5:**
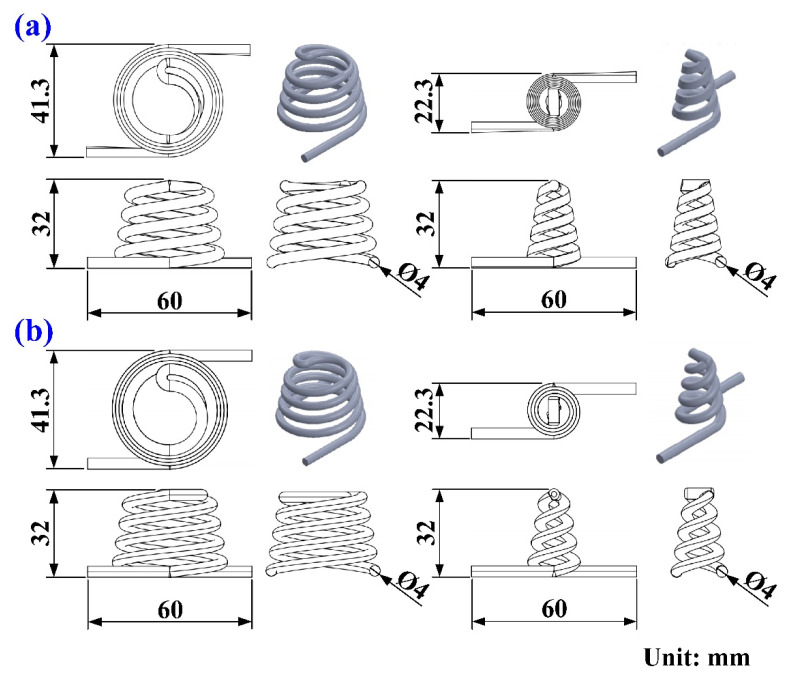
Layouts of (**a**) PCCC and (**b**) CCCC.

**Figure 6:**
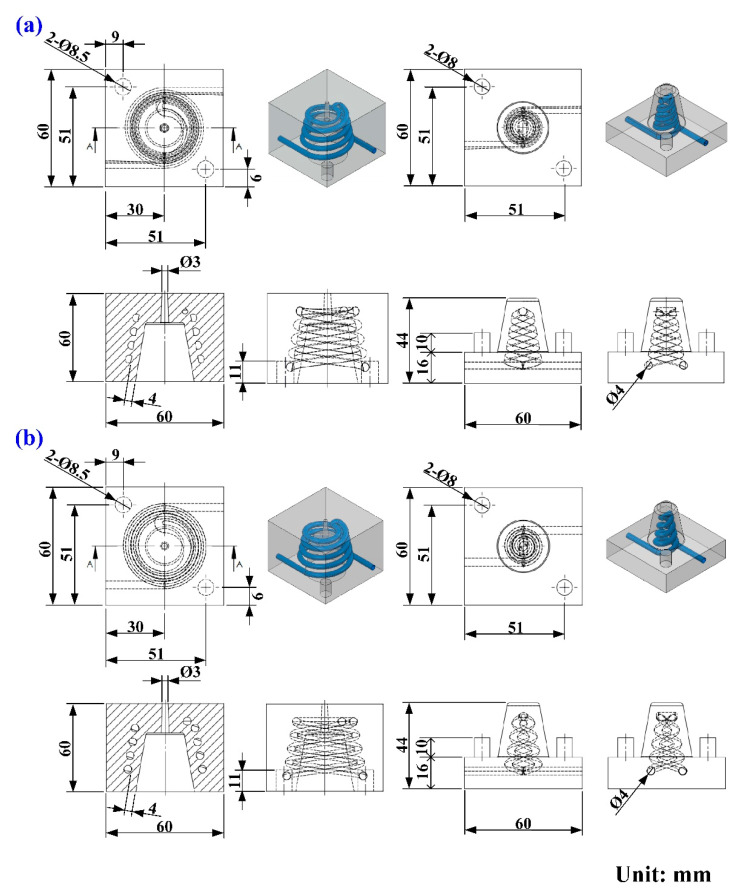
CAD model and dimensions of the wax injection molding tool with (**a**) PCCC and (**b**) CCCC.

Five layout types that can be used for designing a CCC include spiral, zig-zag, scaffold, Voronoi diagram [17], and longitudinal. The spiral CCC was used in this study because of low pressure drop. Figure 5 shows the layout of PCCC and CCCC. On the left-hand side is the cooling channel for the cavity insert. On the right-hand side is the cooling channel for the core insert. Figure 6 shows the CAD model and dimensions of the wax injection molding tool with PCCC and CCCC. The length, width, and height of the core and cavity inserts were all 60 mm. The volume of PCCC and CCCC of the cavity insert was about 2.72 cm^3^, which was about 0.7% of the overall volume of the cavity insert. The cooling channel diameter of PCCC and CCCC was 4 mm. The cross-section areas of PCCC and CCCC were about 8.79 cm^3^ and 12.56 cm^3^, respectively.

**Figure 7:**
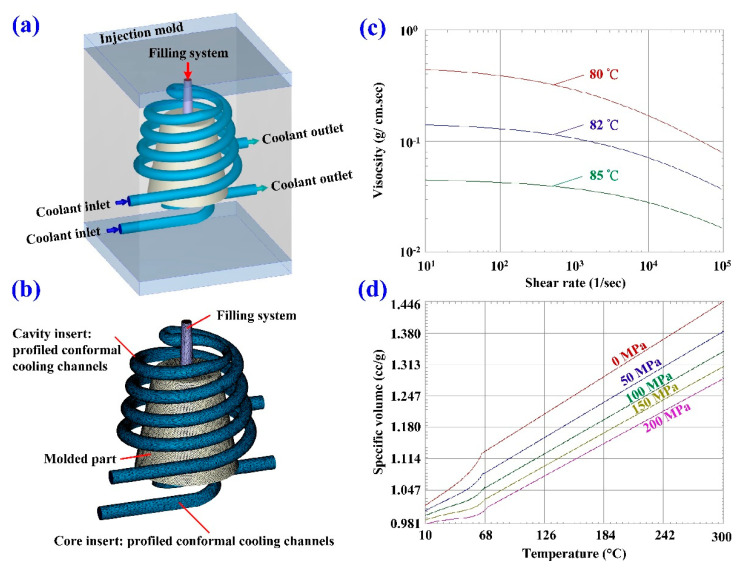
Details of the wax injection molding simulation conditions; (**a**) configuration of the simulation model, (**b**) finite element mesh of profiled conformal cooling channels, filling system, and injection-molded parts, (**c**) viscosity chart, and (**d**) PVT diagram of the molding materials.

**Figure 8:**
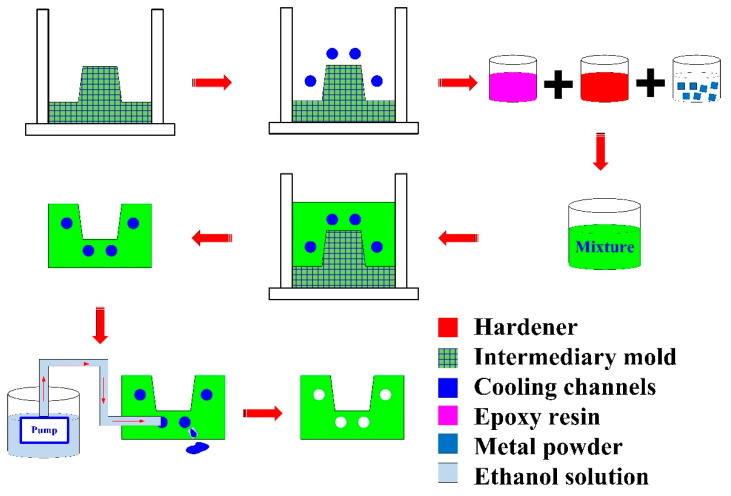
Schematic illustrations of the production process of the wax injection molding tool with cooling channel.

Injection molding simulations were completed with Moldex 3D software. Figure 7 shows the configuration of the simulation model, finite element mesh of profiled conformal cooling channels, filling system and injection-molded parts, viscosity chart, and pressure-specific volume–temperature (PVT) diagram of the molding materials. The relationship of CCC, filling system, injection-molded part, and mold base are illustrated in this figure. The viscosity as a function of the shear rate of the molding material and the pressure, volume, and PVT diagram of the molding material are also illustrated in this figure. In this study, the investment casting wax (K512, Kato Inc., New Taipei city, Taiwan) was used as molding materials. Table 1 shows the main properties of the molding materials. Figure 8 shows the schematic illustrations of the production process of the wax injection molding tool with a cooling channel. In general, additive manufacturing (AM) is capable of manufacturing CCC with complex geometries. Thus, fused filament fabrication [18] was used to print PCCC and CCCC using polyvinyl butyral (PVB) filament [19,20]. The ethanol solution was then used to dissolve the PVB CCC incorporated inside the rapid mold for wax injection molding. Finally, the fabricated rapid mold for wax injection molding was post-cured in a convection oven at 60 °C to obtain the desired mechanical property. It should be noted that the fabricated rapid mold for wax injection molding had limited shrinkage after the post-heat treatment. The main numerical modeling parameters used in the numerical analysis are summarized in Table 2.

**Figure 9:**
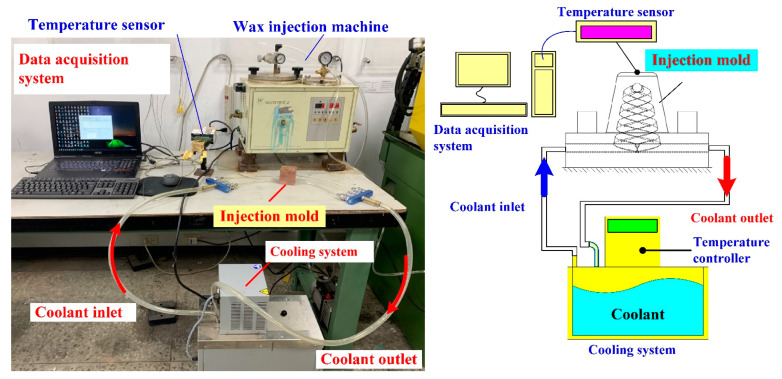
An in-house cooling time measurement system.

The injection molding trials were performed on the low-pressure wax injection molding machine (0660, New Taipei City, Taiwan). The injection mold was held in horizontal position and the molten wax at 82 °C. The demolding temperature of the wax patterns was set at 30 °C via a series of trial runs. An in-house cooling time measurement system was employed to investigate the cooling performance of the rapid mold fabricated, as shown in Figure 9. This system comprises three k-type thermocouples (C071009-079, Cheng Tay Inc., New Taipei City, Taiwan) with a measurement sensitivity of ±1 °C, a mold temperature controller (JCM-33A, Shinko Inc., Nagano, Japan), and a water reservoir with a thermo-electric cooler (TEC12706AJ, Caijia Inc., Taipei City, Taiwan). The k-type thermocouple was embedded in the mold cavity. The other end of the thermocouple was connected to a data acquisition system (MRD-8002L, IDEA System Inc., New Taipei City, Taiwan). The water was employed as the coolant in the cooling system because it has high specific heat capacity compared to oil. The temperature histories of the wax patterns after injection molding were recorded in time intervals of 1 s for determining the cooling time of the injection-molded wax patterns.

## 3. Results and Discussion

### 3.1. Simulation Results

**Figure 10:**
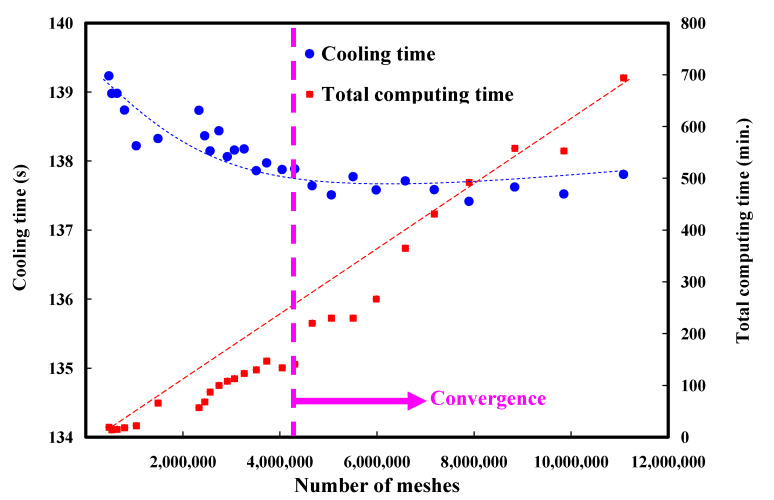
Number of meshes as a function of cooling time and total computing time of numerical analysis.

**Figure 11:**
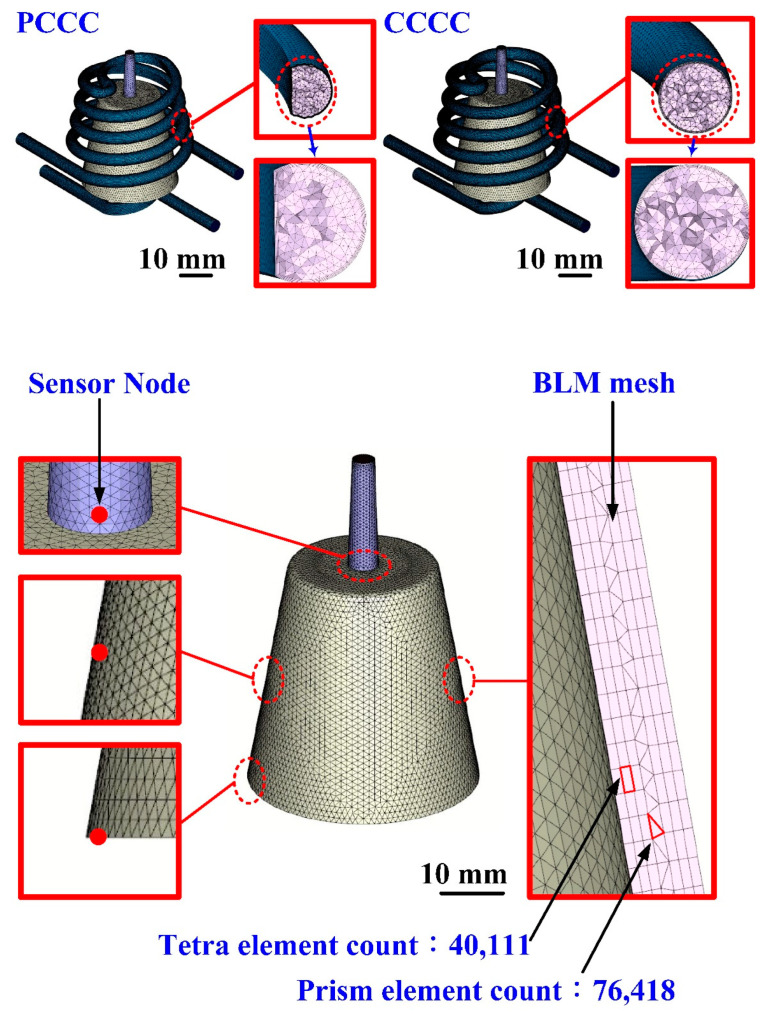
Mesh of the injection-molded part and cooling channels.

**Figure 12:**
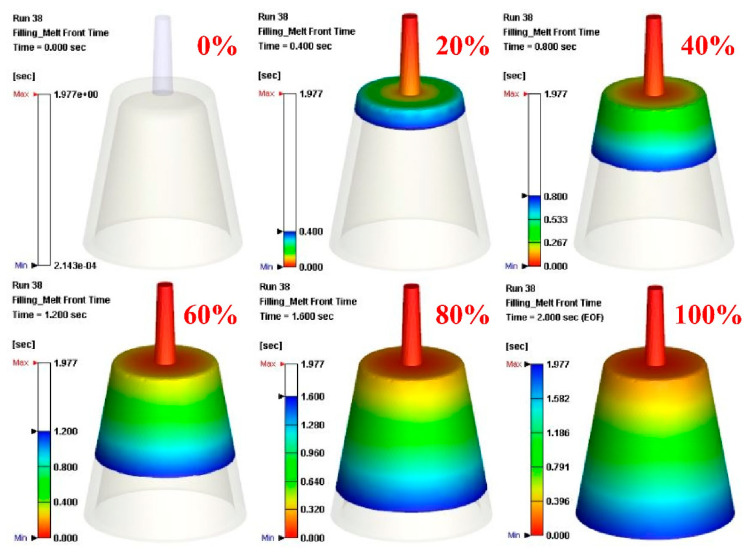
Filling time of the injection-molded part.

In the preliminary stage, the numerical analysis plays an important role in the optimization of the design of the CCC. In general, the 3D solid mesh includes four kinds of meshes, such as prism, tetra, pyramid, and hexahedron [21]. The numbers of nodes for hexahedron, tetra, and pyramid, and hexahedron are 6, 4, and 5, respectively. The mesh generation is a key step in the numerical analysis. Particularly, the boundary layer mesh (BLM) [22] was used in this study to ensure the accuracy of simulation results since it is suitable for simulation models with intricate geometries. Figure 10 shows the number of meshes as a function of cooling time and total computing time of numerical analysis. Generally, a higher number of meshes has a longer total computing time. It should be noted that the cooling time of the injection-molded part was stabilized when the number of mesh element counts exceeded 4,300,000. The cooling time of the injection-molded part was approximately 137–138 s. Therefore, the mesh element count of 4,300,000 seems to be the optimum number of meshes based on both total computing time and cooling time. Figure 11 shows the mesh of the injection-molded part and cooling channels. The mesh of the product was composed of both tetra and prism. The element counts of the tetra and prism were about 40,111 and 76,418, respectively. The wax injection molding process includes three stages: filling, cooling, and ejection stage. In the wax injection molding simulation, the melt front time result showed the position of the melt front with respect to time during the filling stage. Figure 12 shows the filling time of the product. Note that no weld lines or air traps of the injection-molded part were found, meaning that the simulation results showed that the filling process was smooth with the filling time of about 2 s.

**Figure 13:**
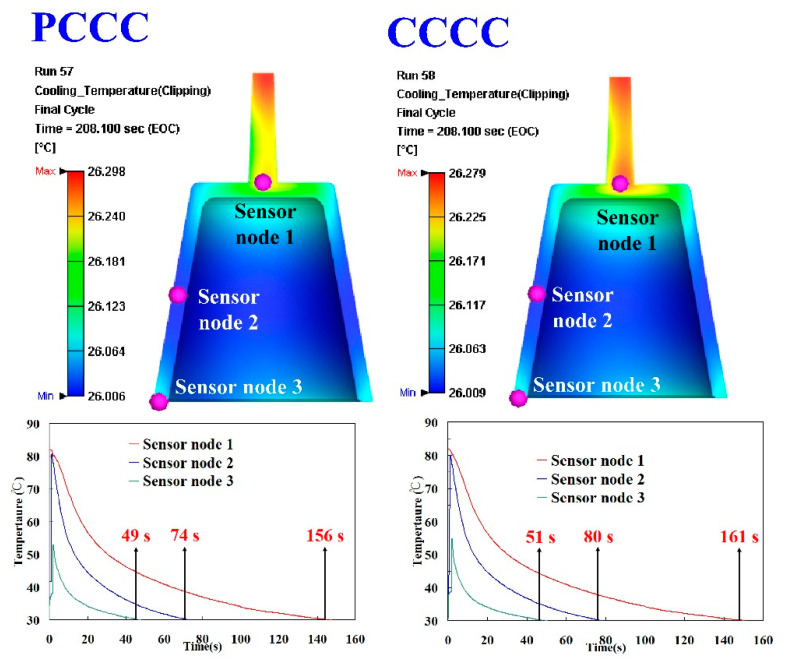
Simulation results of the cooling time of the injection-molded part for the injection mold fabricated by epoxy resin added with 39 vol.% Al powder.

**Figure 14:**
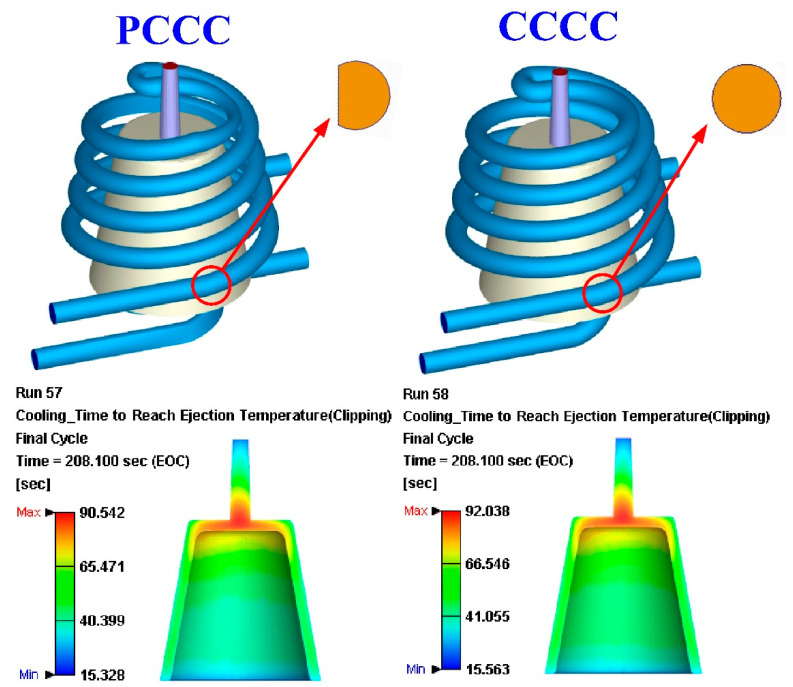
Simulation results of the temperature difference of the injection-molded part of the injection mold fabricated by epoxy resin added with 39 vol.% Al powder.

In this study, three sensor nodes were installed at the top, middle, and end of the wax pattern. The cooling time of the wax pattern means the time to reach the ejection temperature. Figure 13 shows the simulation results of the cooling time of the injection-molded part of the injection mold fabricated by epoxy resin added with 39 vol.% Al powder. As can be seen, the cooling time was shortened from 161 s to 156 s. Figure 14 shows the simulation results of the temperature difference of the injection-molded part of the injection mold fabricated by epoxy resin added with 39 vol.% Al powder. It was observed that the temperature variation of the wax pattern at the end of cooling between 92 °C and 15.6 °C was reduced to the range between 90.5 °C and 15.3 °C. The simulation results showed that the cooling performance of the PCCC was better than CCCC. This result means the PCCC gave higher heat transfer from the mold cavity than CCCC since the PCCC maintained a more uniform and steady cooling performance of the wax pattern than CCCC. Thus, the injection mold with PCCC helps to increase the productivity of the wax patterns in the IC industry.

### 3.2. Fabrication of Injection Molds

**Figure 15:**
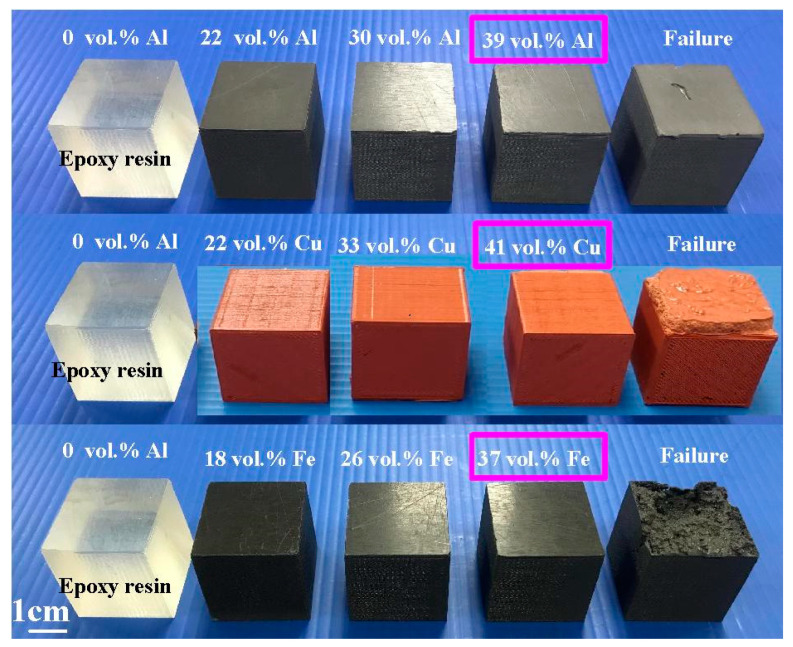
Experimental results of the upper limit of fillers.

To study the upper limit of the vol.% of fillers that can be added to the base material, the vol.% of these four powders was varied from 10 to 95 vol.%. Figure 15 shows the experimental results of the upper limit of fillers. The vols.% of Al, Cu, and Fe powders for the failed specimens were about 41 vol.%, 43 vol.%, and 39 vol.%, respectively. Based on both manufacturability and formability of the test samples, the upper limits of the vol.% of Al, Cu, and Fe powders were found to be approximately 39 vol.%, 41 vol.%, and 37 vol.%, respectively.

**Figure 16:**
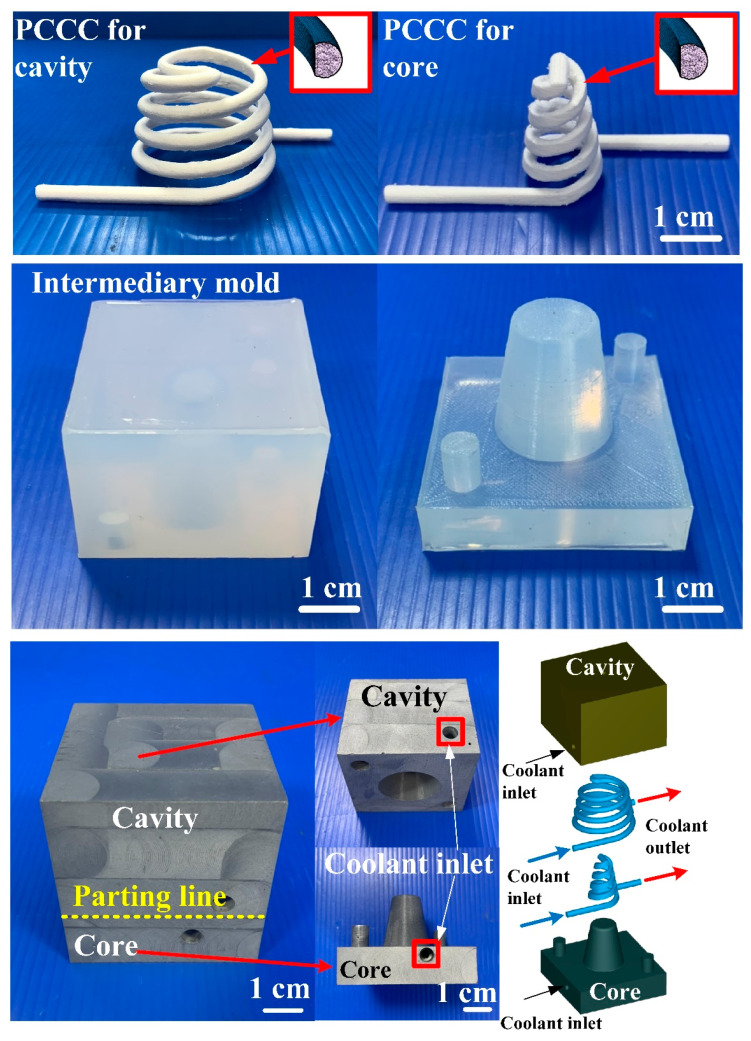
Injection mold fabricated with commercial material.

**Figure 17:**
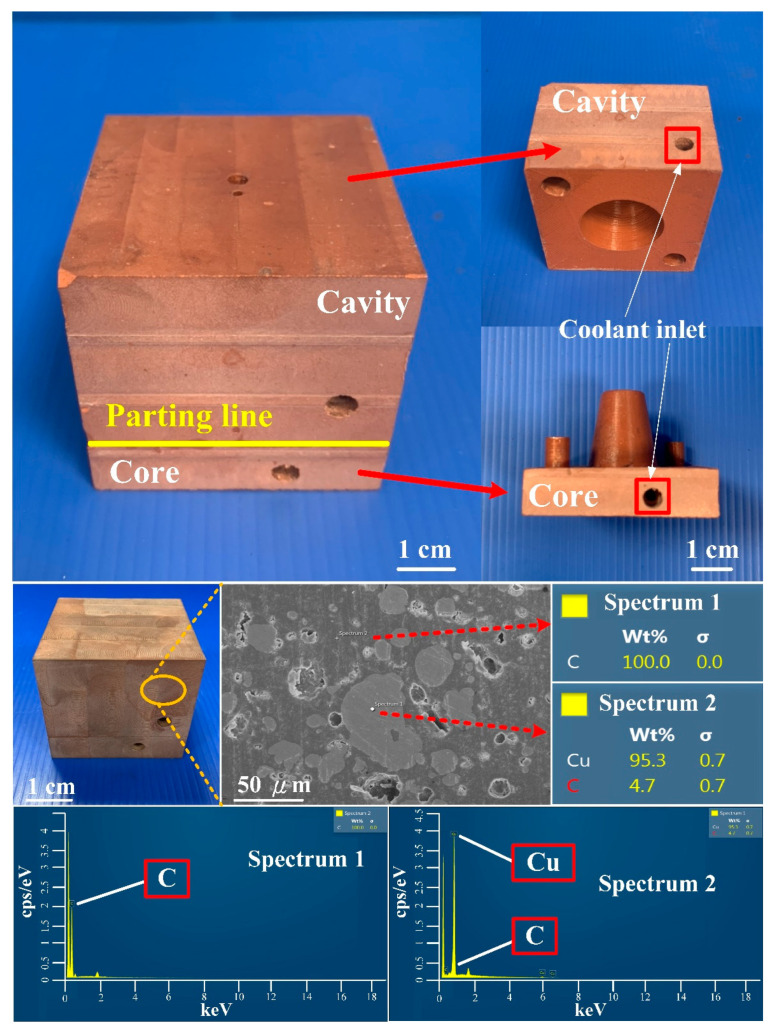
SEM micrographs and chemical composition of wax injection mold fabricated by epoxy resin added with 41 vol.% Cu powder.

**Figure 18:**
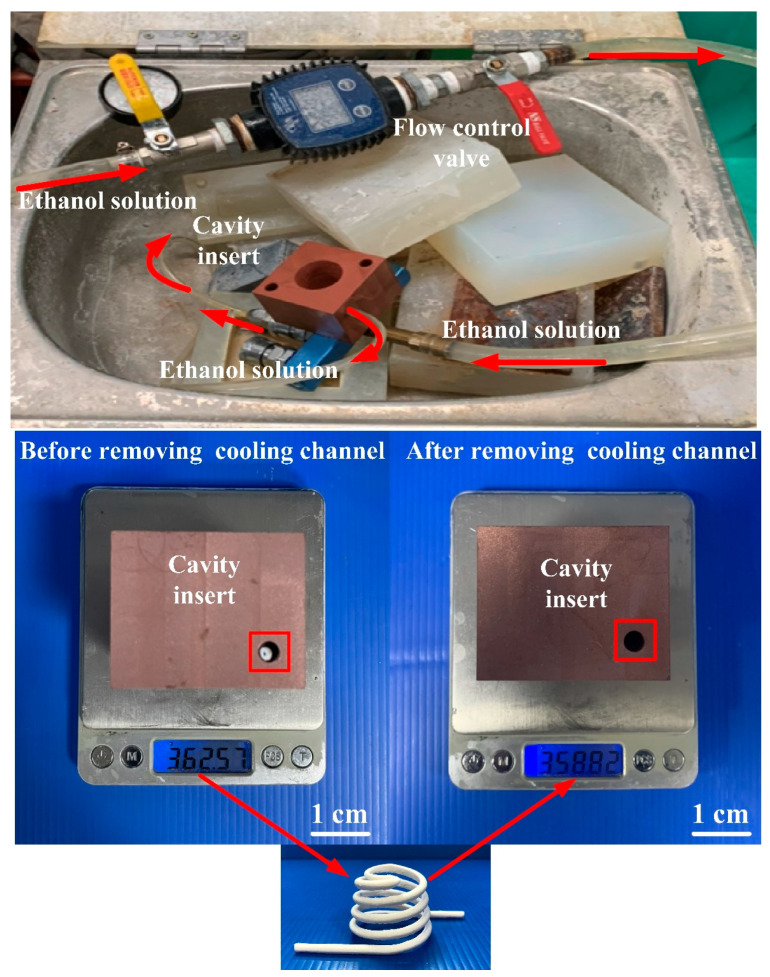
Wax injection mold before and after removing PCCC.

**Figure 19:**
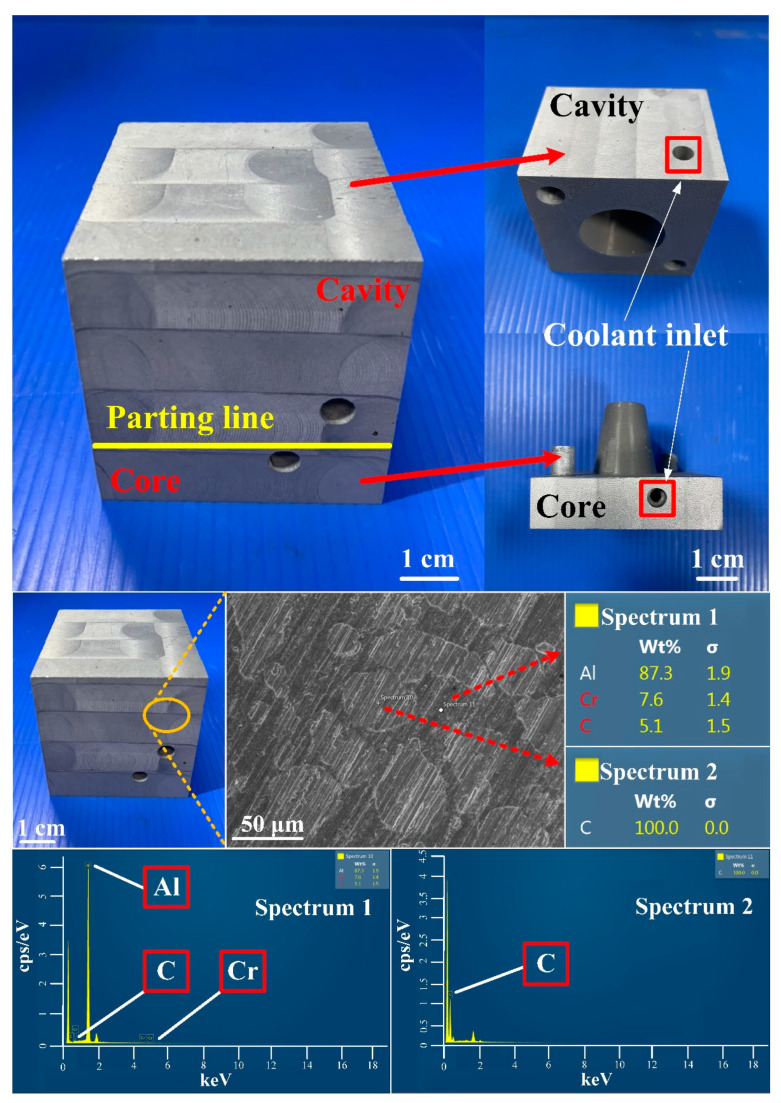
SEM micrographs and chemical composition of wax injection mold fabricated by epoxy resin added with 39 vol.% Al powder.

**Figure 20:**
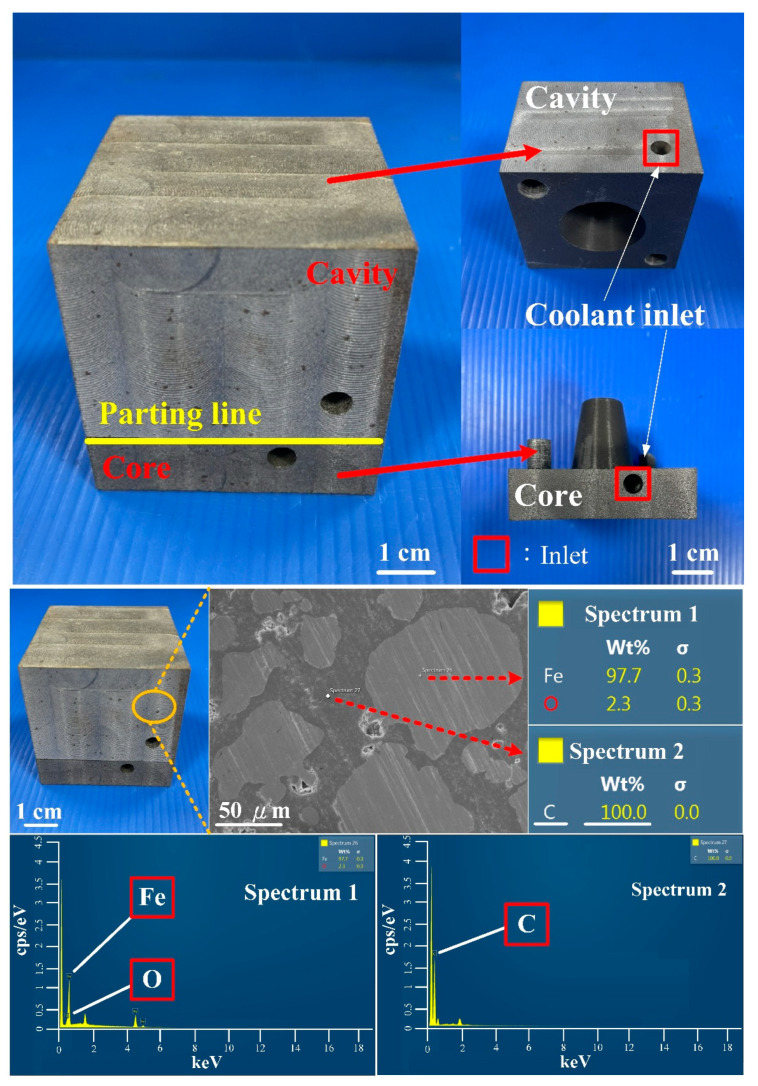
SEM micrographs and chemical composition of wax injection mold fabricated by epoxy resin added with 37 vol.% Fe powder.

Figure 16 shows the injection mold fabricated with commercial material. Figure 17 shows the SEM micrographs and chemical composition of the rapid mold fabricated by epoxy resin added with 41 vol.% Cu powder. The weight of the injection mold with PCCC was 362 g. After removing the PCCC, the weight of the injection mold without PCCC was only 358 g since the weight of the PCCC was 3.75 g. Figure 18 shows the wax injection mold before and after removing PCCC. This result showed that the profiled CCC inside the rapid mold could be removed with ethanol solution completely. Therefore, a rapid mold with PCCC or CCCC can be fabricated by rapid tooling technology. Figure 19 shows the SEM micrographs and chemical composition of the wax injection mold fabricated by epoxy resin added with 39 vol.% Al powder. Figure 20 shows the SEM micrographs and chemical composition of the wax injection mold fabricated by epoxy resin added with 37 vol.% Fe powder.

**Figure 21:**
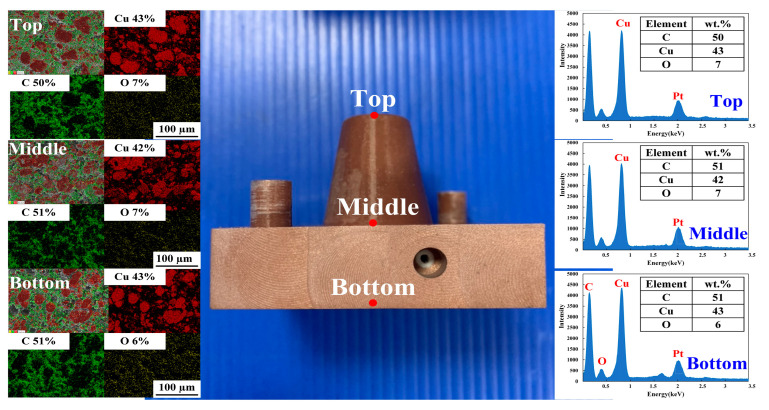
Precipitation analysis of the core insert fabricated by epoxy resin added with Cu powder.

To investigate the precipitation of the fillers in the rapid mold, the EDS analysis was carried out in this study. Figure 21 shows the precipitation analysis of the injection mold fabricated by epoxy resin added with Cu powder. As can be seen, the Cu element content at the top, middle, and bottom of the core inserts were approximately 43%, 42%, and 43%, respectively. The percentages of Cu at the top, middle, and bottom of the ERM were similar, showing that the added Cu powder could be distributed uniformly in the injection mold.

### 3.3. Evaluation of Injection Molds Using Wax Injection Molding

**Figure 22:**
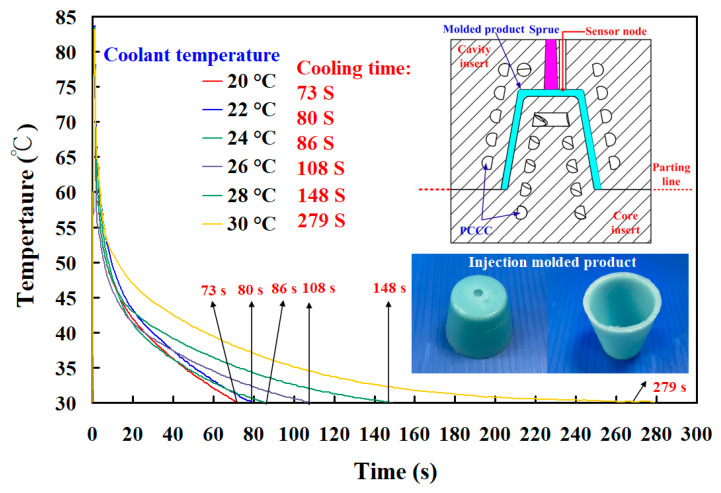
Cooling time of the wax pattern fabricated with epoxy resin added with 39 vol.% Al powder.

Injection molding involves four distinct stages of filling, packing, cooling, and ejecting, which is one of the promising manufacturing methods in the current industries. Figure 22 shows the cooling time of the wax pattern fabricated with epoxy resin added with 39 vol.% Al powder. It was observed that the cooling time of the wax pattern was about 73 s, 80 s, 86 s, 108 s, 148 s, and 279 s when the coolant temperature was 20 °C, 22 °C, 24 °C, 26 °C, 28 °C, and 30 °C. Two phenomena were found. One is that the cooling time of the wax pattern was affected by the coolant temperature. The other is that the cooling time of the wax pattern was shorter when the coolant temperature was lower. It is interesting to note that the same phenomena were also found in the wax patterns fabricated with epoxy resin added with 41 vol.% Cu powder and 37 vol.% Fe powder.

**Figure 23:**
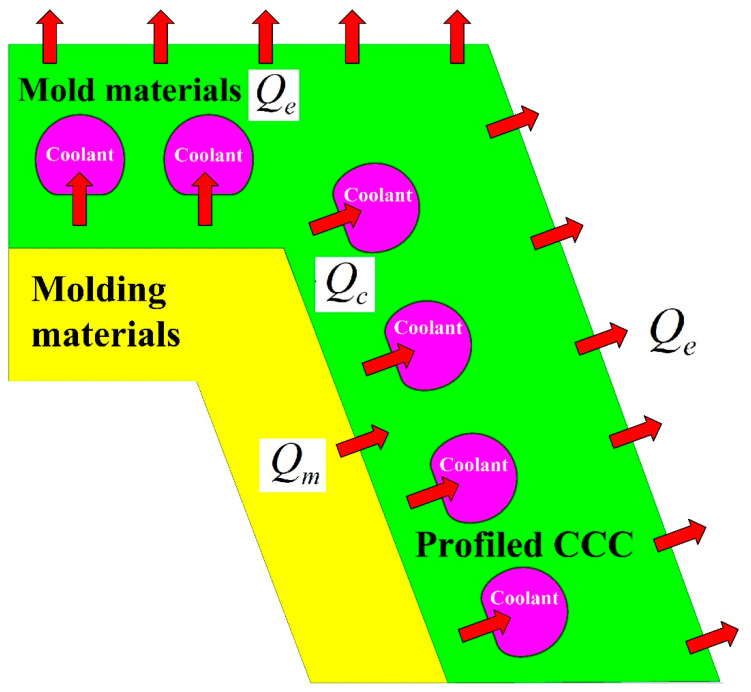
Schematic illustration of the heat fluxes of PCCC during the cooling stage.

In general, the cooling stage is a sophisticated heat transfer process in the injection molding process. The aim of PCCC is to maintain a uniform and steady cooling performance of the wax pattern after wax injection molding. Figure 23 shows the schematic illustration of the heat fluxes of PCCC during the cooling stage. $Q_{m}$, $Q_{c}$, and $Q_{e}$ stand for the heat transfer rate from the molding material to the mold materials, heat transfer rate from the mold material to the coolant, and heat transfer rate from the mold material to the air, respectively [23]. To study the heat transfer process, the cycle-averaged temperature distribution represented by the steady-state Laplace heat conduction equation was widely employed to simplify the cooling process during the cooling stage [24].

**Figure 24:**
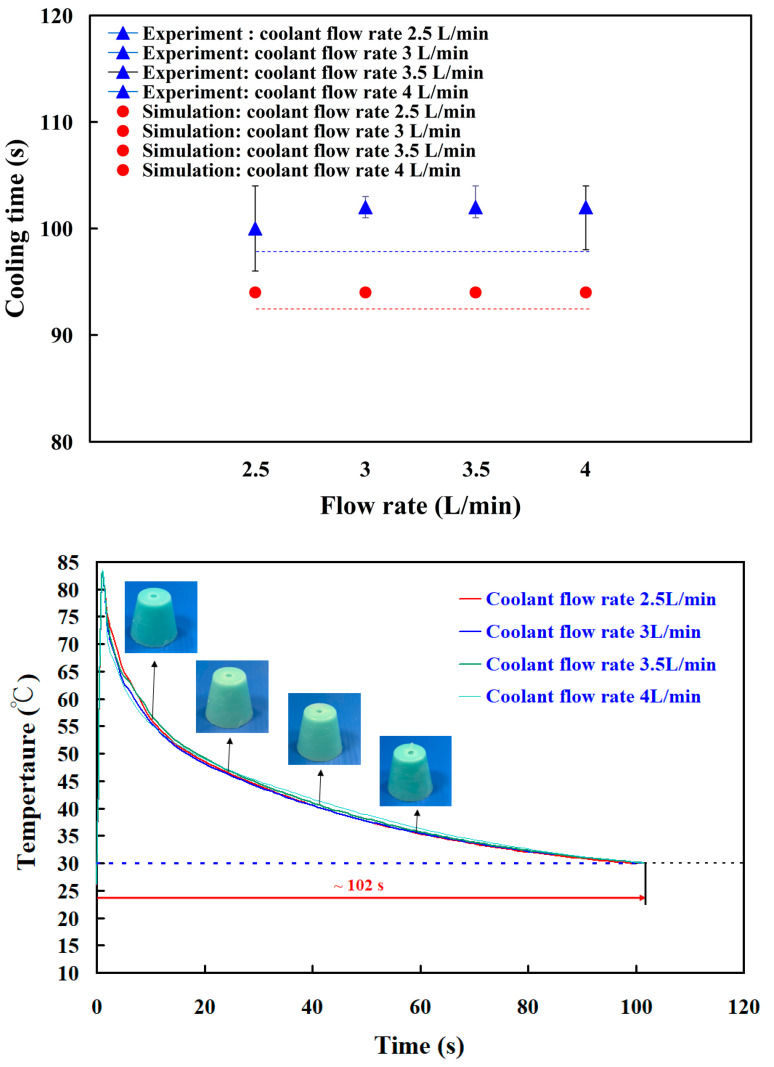
The cooling time of the wax pattern as a function of flow rate.

In practice, the coolant flow rate is an important issue on the cooling efficiency for the rapid mold. Note that the turbulent flow provides three to five times as much heat transfer as laminar flow. In this study, four different coolant flow rates were used, i.e., 2.5 L/min, 3 L/min, 3.5 L/min, and 4L/min. The Reynolds number for four different coolant flow rates was about 14,430, 16,640, 17,650, and 19,250, respectively. The coolant flow reaches a complete turbulent flow when the Reynolds number exceeds 10,000. To study the effects of coolant flow rates on the cooling performance, a series of test runs was performed. Figure 24 shows the cooling time of the wax pattern as a function of flow rate. This results showed that changing the coolant flow rate had no significant effect on the cooling time of the injection-molded wax pattern. As can be seen, the experimental results are consistent with the results predicted by the numerical analysis with the error rate being about 8–10%. This discrepancy was mainly related to the inconsistency in the boundary conditions between the simulation software and experiment.

**Figure 25:**
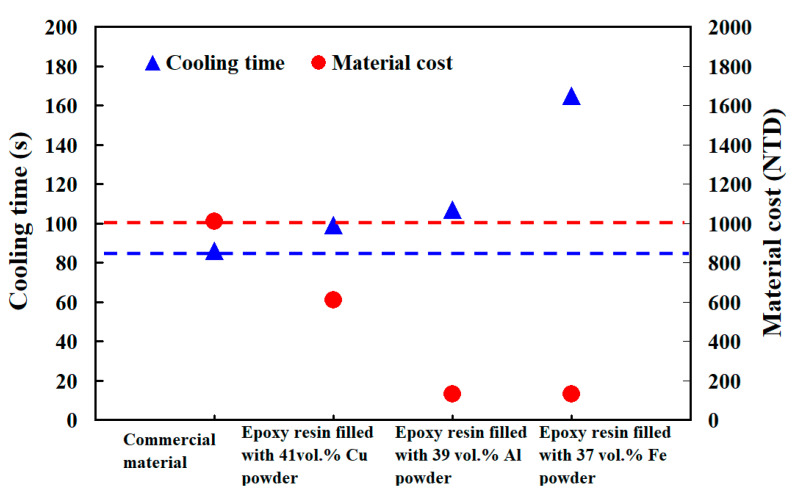
Four different kinds injection molds of material cost and cooling time of the wax pattern.

To understand the differences in the cooling time between the numerical simulation and experiment, the actual cooling time was measured by wax injection molding. Figure 25 shows the four different kinds injection molds of material cost and the cooling time of the wax pattern. It is important to note that the material costs of injection molds fabricated with commercial material, epoxy resin added with 41 vol.% Cu powder, epoxy resin added with 39 vol.% Al powder, and epoxy resin added with 37 vol.% Fe powder were about NTD 1010, 610, 132, and 130, respectively. The cooling times of the wax patterns manufactured by injection molds fabricated from commercial material, epoxy resin added with 41 vol.% Cu powder, epoxy resin added with 39 vol.% Al powder, and epoxy resin added with 37 vol.% Fe powder were about 86, 99, 107, 165 s, respectively. Note that rust was observed in the PCCC wall for the injection mold fabricated by epoxy resin added with 37 vol.% Fe powder, which affected the heat transfer during cooling stage significantly. The cooling performance of injection molds fabricated by epoxy resin added with 41 vol.% Cu powder could reach 88% of that of the injection mold fabricated with commercial material. It should be noted that the material cost of making the injection mold was only about 60% of that of the injection mold fabricated with commercial material. The cooling performance of injection molds fabricated by epoxy resin added with 39 vol.% Al powder could reach 80% of that of the injection mold fabricated with commercial material. However, the material cost of making the injection mold was only about 87% of that of the injection mold fabricated with commercial material. Based on both material cost of making the injection mold and cooling time of the wax pattern, it was found that the epoxy resin added with 41 vol.% Cu powder seems to be an empirical material formulation. According to the experience from mass production of wax patterns, the unit cost of the wax patterns decreases as a result of shorter cooling time. Thus, this empirical material formulation can be used for wax injection molding applications economically because rapid manufacturing is the goal. The potential benefits are cost saving and lead-time reduction compared to conventional mold steel because the developed epoxy-based rapid mold is capable of duplicating batches of wax patterns swiftly and economically.

In general, the manufacture of turbine blade by the lost-wax casting is very common. The fabricated injection mold can be used for low-volume production of wax patterns in the IC industry [25]. It could be concluded that the findings of this study are very practical and provide the greatest application potential in the foundry because the mass production of a wax pattern is a key process in the IC industry. In this study, water was employed as the cooling medium in the cooling system. An alternative coolant such as compressed gas, ultrafine bubble, or cold stream can also be employed to investigate the difference in the cooling performance. It should be noted that the fabricated injection mold can also be employed to plastic injection molding [26,27]. The heat transfer was affected significantly by the deposition of the limescale in the wall of the cooling channel during mass production using injection molding. Investigation of effects of limescale thickness on the heat transfer in the injection molding process is an important research issue. In addition, the mechanical property of the epoxy-based rapid mold was limited due to intrinsic material properties. Some reinforcement fillers, such as alumina, boron nitride, mullite ceramic, silica, molybdenum disulfide, aluminum oxide, wollastonite, zircon, glass fibers, or silicon nitride, can also be suggested to add in the epoxy-based rapid mold to further improve the mechanical property of the injection mold. In this study, the PVB polymer was used to fabricate PCCC. Alternative materials, such as wax, polylactic acid, or acrylonitrile butadiene styrene, can also be used to fabricate PCCC. In this study, the developed empirical material formulation was used to manufacture a wax injection mold. It is important to note that the developed empirical material formulation can be further applied to plastic extrusion [28], gravity die casting, hot stamping, blow molding, metal injection molding, powder metallurgy, die casting, injection compression molding [29], or rotational molding [30]. These issues are currently being investigated and the results will be presented in a later work.

## 4. Conclusions

In the precision foundry industry, reducing the cooling time of a molded wax pattern is the key for mass production of wax patterns with sophisticated geometries. The epoxy-based rapid mold provides promising advantages in reducing the lead time and costs for rapid mold making. The cooling performance depends on the layout of the cooling channels and the thermal characteristics of the mold materials. It is well-known that the productivity of wax patterns was not good enough for a wax injection molding tool with straight-drilled cooling channels. The main purpose of this study was to characterize the epoxy-based rapid mold with PCCC. The results of this study may provide the greatest application potential in the IC industry since the cooling time reduction provides a significant impact on the production cost in the mass production of metallic components using IC. Based on the results discussed in this study, the following conclusions could be drawn:

(1) The simulation results revealed that the cooling performance of the PCCC was better than CCCC since PCCC maintained a more uniform and steady cooling performance of the wax pattern than CCCC.
(2) A cost-effective empirical material formulation composed of epoxy resin added with 41 vol.% Cu powder was demonstrated in this study. This material formulation provided an injection mold with low manufacturing cost and good cooling performance simultaneously compared to an injection mold fabricated with commercial material.
(3) The cooling performance could reach 88% of that of the injection mold fabricated with commercial material.
(4) The material cost of making the injection mold was only about 60% of that of the injection mold fabricated with commercial material.
(5) The cooling time of the wax pattern was affected by coolant temperature significantly because the cooling time was shorter when the coolant temperature was lower. However, the coolant flow rate had no significant effect on the cooling time of the injection-molded wax pattern.

## Figures and Tables

**Table 1:** Main properties of the casting materials.

Item	Data
Melting point (°C)	80–85
Specific gravity	0.96
Linear shrinkage (%)	0.9–1.0
Poisson ratio	0.17
Penetration	9

**Table 2:** Main numerical modeling parameters used in the numerical analysis.

Parameters	Value
Injection pressure (MPa)	0.06
Melt temperature (°C)	82
Injection mold temperature (°C)	27
Shot volume (cm^3^)	5.48
Holding time (s)	0.1
Demolding temperature (°C)	40

## Data Availability

Not applicable.
